# Ubiquitin Carboxy-Terminal Hydrolase-L1 as a Serum Neurotrauma Biomarker for Exposure to Occupational Low-Level Blast

**DOI:** 10.3389/fneur.2015.00049

**Published:** 2015-03-16

**Authors:** Walter Carr, Angela M. Yarnell, Ricardo Ong, Timothy Walilko, Gary H. Kamimori, Uade da Silva, Richard M. McCarron, Matthew L. LoPresti

**Affiliations:** ^1^Center for Military Psychiatry and Neuroscience, Walter Reed Army Institute of Research, Silver Spring, MD, USA; ^2^U.S. Army Special Forces Command, Fort Bragg, NC, USA; ^3^Applied Research Associates, Inc., Littleton, CO, USA; ^4^NeuroTrauma Department, Naval Medical Research Center, Silver Spring, MD, USA

**Keywords:** biomarker, blast, military, neurotrauma, breacher

## Abstract

Repeated exposure to low-level blast is a characteristic of a few select occupations and there is concern that such occupational exposures present risk for traumatic brain injury. These occupations include specialized military and law enforcement units that employ controlled detonation of explosive charges for the purpose of tactical entry into secured structures. The concern for negative effects from blast exposure is based on rates of operator self-reported headache, sleep disturbance, working memory impairment, and other concussion-like symptoms. A challenge in research on this topic has been the need for improved assessment tools to empirically evaluate the risk associated with repeated exposure to blast overpressure levels commonly considered to be too low in magnitude to cause acute injury. Evaluation of serum-based neurotrauma biomarkers provides an objective measure that is logistically feasible for use in field training environments. Among candidate biomarkers, ubiquitin carboxy-terminal hydrolase-L1 (UCH-L1) has some empirical support and was evaluated in this study. We used daily blood draws to examine acute change in UCH-L1 among 108 healthy military personnel who were exposed to repeated low-level blast across a 2-week period. These research volunteers also wore pressure sensors to record blast exposures, wrist actigraphs to monitor sleep patterns, and completed daily behavioral assessments of symptomology, postural stability, and neurocognitive function. UCH-L1 levels were elevated as a function of participating in the 2-week training with explosives, but the correlation of UCH-L1 elevation and blast magnitude was weak and inconsistent. Also, UCH-L1 elevations did not correlate with deficits in behavioral measures. These results provide some support for including UCH-L1 as a measure of central nervous system effects from exposure to low-level blast. However, the weak relation observed suggests that additional indicators of blast effect are needed.

## Introduction

Repeated exposure to low-level blast is a characteristic of a few select occupations and there is concern that such chronic exposure presents risk for traumatic brain injury (1). The hypothesized injury to the brain from repeated blast overpressure may be analogous to the mounting recognition of problems from multiple sports-related concussions and sub-concussive impacts (2, 3). Observations are similar for symptomology associated with sports-related concussion and symptomology associated with exposure to repeated blast (4); however, there is a key difference from sports-related concussion, or even sub-concussive impacts, in that there is no physical impact to the head from routine occupational blast exposure. This difference has prompted a unique line of research on effects from occupational exposure to low-level blast. A challenge in this research has been the need for improved assessment tools, particularly objective markers suitable for field settings, where transient acute effects are most likely to occur (5). The objective of this report is to evaluate the utility of a blood-based neurotrauma biomarker, ubiquitin carboxy-terminal hydrolase-L1 (UCH-L1), as a marker of neurological effects from occupational blast exposure in humans.

Human exposure to blast can occur in a military combat environment with acute effects on the brain and resulting clinical conditions. Occupational exposure to blast also occurs outside the combat environment in military and specialized law enforcement training. For military personnel, this includes training in artillery/mortar firing, grenades and distraction devices (e.g., flash bangs), a variety of shoulder-fired weapons (e.g., high powered rifles), and explosive breaching. For non-military personnel, specialized law enforcement units may not train with artillery or mortar weaponry, but they do train with explosive breaching. In the case of breaching, operators construct an explosive charge and detonate it while at relatively close distances. This tactical technique is used to create a breach in a locked door or wall to afford rapid entry to a secured structure. To employ explosive breaching effectively, personnel train with explosive charges resulting in hundreds or thousands of exposure to low-level blast. In the case of those designated as instructors, who have the responsibility of training others, routine exposures to blast can number in the tens of thousands. Safety protocols for such exposure typically stipulate that blast overpressure for personnel should not exceed 4 psi in any single event. Those protocols are followed through *a priori* estimation of overpressure levels, which has been a more practical method than actual measurement of pressure experienced by operators. It was not until relatively recently that pressure sensor technology could be worn by individuals without interfering with operations.

The 4 psi safety threshold is based on the force observed to rupture the unprotected human tympanic membrane. The threshold for an overpressure injury to the human central nervous system (CNS) is unknown due to the lack of exposure magnitude data available for acute events resulting in clinically diagnosed injuries. The potential for that threshold to change with multiple low-level exposures, or tens of thousands of such exposures, has only recently been considered. There is no known lower limit of exposure magnitude that can be considered irrelevant in the context of many repeated exposures. For the purposes of the study reported here, “low-level blast” is not defined as a quantified range of overpressure values but rather as blast used in standardized training protocols, settings in which clinical diagnosis of primary blast injury does not occur. Now that technological advances make it feasible to measure individualized exposures to occupational blast, there is an opportunity to empirically evaluate effects that are suspected to follow from repeated low-level blast exposure in humans. The challenge is identifying effective outcome measures.

A survey of symptomology among professional breachers showed elevated rates of self-reported headache, sleep disturbance, and working memory impairment, among other symptoms (4). This symptomology was also reported by breachers in the survey to interfere with daily function but it was not concomitant with any clinical diagnosis of head injury, only with repeated exposure to blast. Further, the greater the number of reported blast exposures, the more frequent and severe the reported symptomology. A parallel association of symptomology and degree of blast exposure is seen within a sample of military personnel who have been diagnosed with concussion (6). This pattern of concussion-like symptomology and association with occupational blast raises the concern that such repeated exposures, even at low-level magnitude, entail risk to the CNS. To date, there have been several efforts to study the relation of occupational blast exposure and CNS damage in humans (7–9). Those studies used more objective assessments than self-report of symptoms, including measures of sleep disruption, vestibular stability, neurocognitive performance, and serum-based neurotrauma biomarkers.

Kelley et al. (7) equipped 18 military breachers with wrist-worn actigraphy units for the duration of a 2-week explosive breaching training protocol. Actigraphy uses accelerometers to continuously record limb motion, which reliably reflects periods of activity and periods of sleep and its use for assessing sleep patterns in normal, healthy adult populations is endorsed by the American Academy of Sleep Medicine (10). Although subjects reported greater daytime sleepiness in conjunction with explosive breaching, actigraphy assessments did not reveal a corresponding change in sleep/wake data. Baker et al. (8) used a variety of empirically validated vestibular assessments and neurocognitive assessments to examine 14 civilian law enforcement breachers completing a 10-day explosives techniques instructor course. The behavioral assessments were administered by physicians and trained technicians and were augmented by a computer-based neurocognitive assessment. Overall, these assessments did not show deficits associated with breaching blast exposure. The most recent of these studies (9) employed serum-based neurotrauma biomarkers alongside computer-based neurocognitive assessment with 19 soldiers during a 2-week explosive entry course, and reported results showed some association between low-level blast exposure, deficits in neurocognitive performance, and neurotrauma biomarker elevation. However, evidence for that association was isolated to a few individuals and based on composite measures, combining multiple behavioral measures and multiple biomarkers into single outcomes. Each of these studies cited a key limitation from the low number of available subjects and none included individually measured blast overpressure. It is notable that the study employing serum-based neurotrauma biomarkers showed the clearest association between low-level blast exposure and outcome in objective measures (9). The present study expands available evidence by using a relatively large sample size and by measuring the magnitude of blast exposure in conjunction with a concurrent serum-based neurotrauma biomarker.

Ubiquitin carboxy-terminal hydrolase-L1 was the targeted biomarker for evaluation in the present study, given both empirical and observational support in the current literature suggesting a correlation with sub-concussive head impact. A recent review of biomarkers for mild traumatic brain injury in blood identified UCH-L1 as a candidate with empirical support (11), and a study of UCH-L1 in sub-concussive sports-related head impacts showed UCH-L1 increased with participation in collegiate level American football (12). Evidence of epigenetic changes to the ubiquitin pathway among patients with blast-related mild traumatic brain injury also suggests that there may be changes in the UCH-L1 proteomic marker following exposure to blast (13). Furthermore, increases in serum UCH-L1 have been reliably observed <1 h following trauma, even among persons with mild injury severity and no Glasgow Coma Scale deficit (14). This rapid change was also observed in the study of UCH-L1 and sports-related sub-concussive head impacts (12). Similar to observations of rapid appearance of UCH-L1 after brain injury, UCH-L1 has also been observed to rapidly diminish in serum after concussion in a military sample (Carr et al., under review) as well as after more severe injury (15). The low-level blast exposure study by Tate et al. (9) employed serum-based neurotrauma biomarkers and described biomarker “spikes” in their repeated measures paradigm. These observations support the likelihood that changes in UCH-L1 level following mild insult to the brain would be transient and could serve to reflect day-to-day variations in environmental exposure.

The prospective observational study reported here examined daily sampling of peripheral blood for UCH-L1 changes in conjunction with measurement of repeated exposure to low-level blast. Blast-related effects were evaluated from normal healthy individuals in field research paradigms rather than a clinical setting. As such, effects were believed to be inherently small and were expected to be subject to individual differences and factors beyond the control available in laboratory experimental paradigms. Nonetheless, exposure to blast is hypothesized to result in acute elevations in serum UCH-L1 concentrations, even in the relatively low-level blast experienced in training paradigms for explosive breaching. However, this low-level blast environment does not typically result in acute diagnosable injury, so the magnitude of an association between UCH-L1 and low-level blast exposure is expected to be limited. This study maximized the hypothesized effect by enrolling participants with a history of blast exposure, which was expected to magnify effects in a manner similar to multiple sports-related concussions.

## Materials and Methods

### Participants

The protocol for this study was reviewed and approved by the Naval Medical Research Center and Walter Reed Army Institute of Research Institutional Review Boards. Across three separate sites, 108 male, active duty military personnel provided written informed consent to participate in this study while engaged in training as either students or instructors. Prior to initiation of the study, leadership from each site gave authorization for subject recruitment and research activity during 2 weeks of standardized training exercises. Training programs at all three sites were designated as advanced courses, requiring prior experience in explosive breaching as prerequisite for entry. Therefore, all participants had prior exposure to low-level blast overpressure. All individuals who were enrolled as students or designated as instructors for the training programs were eligible to participate in the study. To maintain group integrity and avoid isolating individuals during training, no exclusion criteria were used.

During study enrollment, research participants provided self-report of health history information, including history of blast exposure and head injury, as well as experience with explosive breaching. In data collection on training days with blast exposure, participants were instrumented with two pressure sensors to record blast parameters for each individual during training exercises. At the end of every training day, regardless of blast exposure, participants had blood drawn, and completed behavioral measures of symptomology, postural stability, and computer-based neurocognitive performance. Participants also wore wrist actigraphs continuously throughout the 2-week period to monitor sleep. At the time of this study, no females participated in these training programs. Participants were compensated $25 per blood draw, as authorized under Department of Defense policy for research participation by military personnel on duty (DoDI 3216.02, 2011). Participant characteristics for the three sites are listed in Table 1.

**Table 1:** **Demographic characteristics of study participants**.

	Site 1	Site 2	Site 3	Total
*N*	47	28	33	108
Age (years old)	25 (SD = 3.8)	33 (5.2)	31 (4.9)	29 (5.9)
Prior head injury (yes/no)	23% (of sample)	41%	42%	34%
Prior blast exposure (yes/no)	75% (of sample)	65%	79%	74%
Prior breaching exposure^a^	40–99	100–199	40–99	40–99

*^a^History of breaching exposure was assessed by a 7-level ordinal scale item with the following response levels for number of explosive breaches experienced: 0, 1–9, 10–39, 40–99, 100–199, 200–399, or 400+. Responses were scored as values 0–6 and the descriptor in the table represents the level that corresponded to the average of scores*.

### Training environments

**Figure 1:**
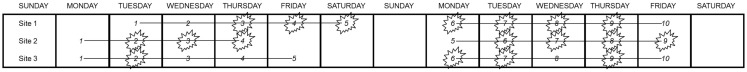
**Representation of training schedule and days of blast exposure across a 2-week period for each of the three study sites**. Training days are labeled with serial Session numbers, with Sites 1 and 3 showing 10 Sessions and Site 2 showing 9 Sessions. Sessions with blast exposure are indicated by a jagged line surrounding the Session serial number. Group-level blast was recorded in 7 of 10 Sessions at Site 1, 7 of 9 Sessions at Site 2, and 4 of 10 Sessions at Site 3.

The three sites were similar in that each site employed breaching blast with dismounted personnel across a 2-week period. The sites trained personnel for different operations and had differences in the frequency and type of explosive charges used. Figure 1 shows a 2-week time period with depiction of training days and blast exposure days for each site. Figure 1 labels training days with serial numbers (“Sessions”). Data are reported according to Session.

### Blast measurement

Overpressure from explosive blast was measured by pressure sensors (micro Data Acquisition System, μDAS; Applied Research Associates, Inc.) mounted on each subject’s helmet above left and right ear cups. The helmet is considered to be the best place to measure pressures in a study of traumatic brain injury because the sensors are close to the brain and the helmet provides a consistent spacing and orientation for the pressure measurements. The system is designed to continuously monitor the environment for changes in ambient pressure and is triggered to record data when a threshold of 0.4 psi is exceeded on either sensor. This threshold was chosen based on the technological specifications of the sensors as well as considerations for signal to noise ratio in the interpretation of sensor data output. For each triggered blast event, the μDAS recorded the time of the event trigger, the peak pressure (maximum overpressure reached during the blast event), and the impulse energy (integral of overpressure over time throughout the blast event). For each day of blast exposure, the number of triggered events, the highest recorded peak pressure, and the highest recorded impulse energy were logged for each participant.

For the analyses in this report, data used for each blast exposure event were the averages of measurements from left and right sensors. The rationale for averaging the measurements from sensors mounted on the left and right sides of the helmet was to address potential confounds introduced by the physical relation between the sensors and blast wave path. For example, two persons equipped with only one helmet-mounted sensor and who are at equivalent distance from an open field blast may yield different recorded pressures if one person’s sensor is exposed to the direct path of the blast wave (reflective pressure) and the other person’s head is turned, putting that sensor at a right angle to the path of the blast wave (incident pressure) (16). A sensor in the shadow of the helmet would likely yield lower pressure magnitude, even though the two individuals may have experienced equivalent levels of pressure.

### Blood collection and UCH-L1 assay

Peripheral blood draw was taken at the end of each training day, which was generally late afternoon and approximately the same time each day. Due to the observational nature of this protocol, collecting research measurements in context of standard military training, the time between blast exposure and blood draw was not subject to control but all measurements were taken within 8 h from the most recent exposure to blast that day (on the days in which blast occurred). Blood was drawn from each subject on each day regardless of blast exposure that day, so some Sessions will reflect UCH-L1 concentration with no blast exposure in the preceding 24 h. Blood samples were processed for serum on site and frozen at −80°C until analysis.

Ubiquitin carboxy-terminal hydrolase-L1 concentration was measured in serum samples by Banyan Biomarkers prototype CL assay, a sandwich ELISA. Samples were assessed in duplicate with 96-well plates coated with a capture antibody specific to UCH-L1. Plates were then washed and a second biomarker-specific antibody (detection antibody) was added, which created the “sandwich.” The detection antibody was conjugated to horseradish peroxidase. The plates were then washed and a substrate was introduced that was converted by the enzyme attached to the detection antibody into a chemiluminescent signal whose intensity was measured electronically. The chemiluminescent signal is directly proportional to the amount of detection antibody and biomarker present.

Determination of the biomarker concentration in a specimen sample is performed by comparing the signal intensity from the unknown sample to a standard curve created from a series of calibrators of known biomarker concentration run on the same 96-well plate as the unknown sample. Specimen samples were tested using the same volume and under the same conditions as those of the calibrators. Quality control samples consisting of a known concentration of biomarker in a representative matrix were included on each plate to allow for confirmation of expected assay performance. Results are reported in picogram per milliliter.

### Additional supporting measures

At the end of each training day, subjects also completed daily assessments of health symptoms, neurocognitive performance, and postural stability. Subjects reported health symptoms using a paper-and-pencil inventory of 32 items associated with concussion (Table 2). This inventory was similar to the Rivermead instrument (17) but with additional items and responses solicited in context of breaching exercises rather than concussion injury. Responses were made on a 5-level Likert-type scale, ranging from 0 (symptom “not experienced at all”) to 4 (“severe problem”).

**Table 2:** **Inventory of health symptoms related to concussion**.

Headaches	Forgetfulness, poor memory	Poor coordination/clumsiness
Feelings of dizziness	Poor concentration	Change in taste/smell
Nausea and/or vomiting	Taking longer to think	Loss of or increased appetite
Easily upset by loud noise	Blurred vision	Difficulty making decisions
Difficulty localizing sound	Easily upset by bright lights	Slowed thinking
Sleep disturbance	Double vision	Difficulty getting organized
Fatigue, tiring more easily	Restlessness	Easily overwhelmed by things
Being irritable, easily angered	Ringing in ears	Light-headedness
Feeling depressed or sad	Pain in ears	Feeling disoriented
Feeling frustrated or impatient	Fullness in ears	Numbness or tingling in body
Feeling anxious or tense	Loss of balance	

Postural stability was assessed by force platform measurement (Biosway Clinical Test for Sensory Integration of Balance, Biodex Medical Systems, Inc., Shirley, NY, USA) (18, 19). Subjects maintained steady posture for 30 s in each of in four conditions: eyes open on firm surface, eyes closed on firm surface, eyes open on dynamic surface (foam pad), and eyes closed on dynamic surface. Each test yields an index score (“sway”) based on the standard deviation of center of gravity deviations in any direction, as sampled every 50 ms.

Computer-based neurocognitive testing was administered with a version of the Automated Neuropsychological Assessment Metrics (ANAM) TBI Battery (20) and the Defense Automated Neurobehavioral Assessment (DANA) (21). The ANAM TBI Battery subtests assess different cognitive abilities and are selected to be sensitive to effects of brain injury (22). This battery has been used elsewhere in research with similar populations and protocols (9; Carr et al., under review). The DANA battery evolved from development work for the ANAM TBI Battery but was designed for application on a handheld device rather than desktop or laptop computer. ANAM and DANA subtests involve visually presented stimuli and computer mouse or stylus responses, which are recorded by the computer and scored for accuracy and response time. Instructions to subjects are to be both “fast and accurate.” These test paradigms are known to show large practice effects (23), yielding large improvements in performance within the first two or three trials. Accordingly, at least two practice trials were conducted before baseline performance to mitigate practice effects and improve sensitivity of these measures.

In addition to the methods described here, additional blood draws and computer-based neurocognitive assessments were completed but they were not administered equivalently at all three sites or were not administered on a daily basis (e.g., pre/post sampling conducted only before and after the 2-week session). Those data are not reported here. Wrist actigraphy was used to monitor sleep patterns to assess potential confounds from sleep disruption and was not a primary outcome measure in this study. Those data are also not reported here.

### Analyses

A criterion for the planned analyses of daily changes in UCH-L1 concentration was that subjects have their blood drawn at least once in the protocol before exposure to blast in order to establish a baseline for each individual. There were three individuals (from Site 2) in the sample of 108 who did not meet this criterion, so they were not included in the analyses.

In the primary analysis of serum UCH-L1 concentrations and correspondence of UCH-L1 change to recorded blast exposures, we compared a specific day in the 2-week training protocols to baseline. This analysis approach was used because the observational design conducted within operational training paradigms was expected to yield differences between individuals in exposure to blast and other relevant environmental factors. The association between UCH-L1 change and blast was expected to be most identifiable on a day in which there was group-level exposure to blast and group-level increase in UCH-L1. This exposure in combination with increases in the marker suggests influence from an environmental factor, mostly likely to be blast. Our approach of comparing a specific training Session to the baseline Session was used to control for confounds, such as days with no blast exposure or exposure for only part of the overall group of subjects.

Baseline levels of UCH-L1 (picogram per milliliter) were expected to be low, entirely or mostly below assay thresholds (i.e., below assay limit of quantification or limit of detection). When UCH-L1 was not detected at the limit of detection (30.0 pg/mL), a replacement value of 15.0 pg/mL was assigned (midpoint between limit of detection and 0) (24). When UCH-L1 was detected but was below limit of quantification (60.0 pg/mL), a replacement value of 45 pg/mL was assigned (midpoint between limit of quantification and limit of detection). Inter-individual variance in absolute concentrations across timepoints was managed by normalizing UCH-L1 concentrations. UCH-L1 values at each timepoint were expressed in terms of percentage of baseline level for each individual (12), thereby affording comparison of relative within-subject change rather than absolute UCH-L1 changes.

Linear regression analyses were used to examine the association between UCH-L1 change and blast magnitude. Characterizing the magnitude of blast can be approached in a variety of methods, especially when there are multiple blasts within the measured period as in these data. Among various accepted measures of blast exposure (e.g., peak pressure, peak impulse, incident pressure, reflective pressure), it is unknown presently which measure (or composite measure) is the most important in the hypothesized effect on the CNS. However, it is known that these different measures will positively correlate with each other. We selected highest recorded peak pressure of the day as the measure to represent blast magnitude. Our rationale was based on the pressure sensors’ high sampling rate and ability to capture peak pressure values, and also the similarity between a timepoint-specific peak and a “traumatic” event, which underlies the hypothesized neurotrauma-induced change in UCH-L1.

The primary analysis, examining if UCH-L1 variance in the Session with the greatest group-level UCH-L1 increase is accounted for by blast magnitude, was supplemented with two secondary analyses. One secondary analysis examined the Session with greatest blast magnitude for a reflection in UCH-L1 concentration. The other secondary analysis examined UCH-L1 on the final day of blast exposure, regardless of group-level UCH-L1 increase or blast magnitude. These analyses were considered secondary because they do not address the question of UCH-L1 increase in relation to blast as clearly as the primary analysis. For normal healthy individuals, it is not yet demonstrated that UCH-L1 will reliably increase following blast, even for the occasion of a relatively high levels of blast in a training paradigm. Assessment of UCH-L1 on the final day of multiple days with repeated blast exposure may reflect a cumulative exposure to blast (i.e., multiple sub-concussive blast events), rather than the acute effect hypothesized for this study. It may be that a single low-level environmental exposure event may not yield acute neurotrauma but, instead, predispose the CNS to be more vulnerable to neurotrauma in a subsequent low-level environmental exposure event. In such case, a transient marker like UCH-L1 may reflect an increase after multiple events rather than a single sub-concussive event but that was outside the scope of this report.

In other supporting analyses, we examined reversals of UCH-L1 increase and correspondence between UCH-L1 increase and demographic factors. When change in UCH-L1 was observed in our analyses, we compared UCH-L1 from the Session with blast exposure to the first subsequent Session in which there was no blast exposure. This comparison leveraged the relatively rapid decline in UCH-L1 following a mild traumatic event and could offer additional evidence that UCH-L1 elevations observed were associated with blast exposure. Observations of UCH-L1 change may be reflected in individual characteristics of past history of exposure to blast or head injury as well as acute change in symptomology, postural stability, or neurocognitive performance. Observation of behavioral change concurrent with UCH-L1 change would have implications for inferences drawn from these data, but this research paradigm is not associated with any clinical injury and behavioral changes that would be clinically relevant were not expected. We looked for subtle changes in these performance variables, but with the expectation that such changes would not be distinct from variance due to factors other than blast exposure (e.g., performance effort, practice effect).

## Results

In repeated measures of the 108 subjects, 10 Sessions for 2 of the 3 groups and 9 Sessions for the remaining group, a total of 1016 blood samples were taken and assayed for UCH-L1. Three subjects did not meet baseline criterion of no acute blast exposure prior to the first blood draw and therefore were not included in analyses, resulting in a total of 992 samples included in the analysis. Of the 992 UCH-L1 assays, 317 samples (32%) had UCH-L1 at detectable levels and 71 samples (7%) showed UCH-L1 at quantifiable levels, ranging from 60.3 to 277.7 pg/mL. Of the 105 subjects included in the analysis, 18 individuals (17% of the overall sample) showed no detectable UCH-L1 at any Session. Within daily assessment Sessions, the average time between last blast exposure and blood draw was 2 h 39 min, with a minimum case of 28 min and a maximum case of 7 h 17 min.

**Figure 2:**
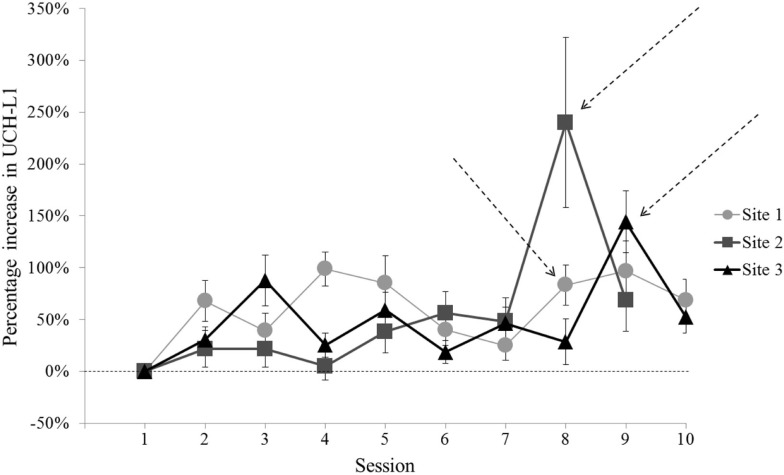
**Normalized UCH-L1 concentrations (relative to individual baseline) for each Session of the study at each of the three sites**. Error bars represent standard error. For two of the three sites, there is a Session in the series that shows a high UCH-L1 level that differs from the next highest Session. [Site 2 Session 8 vs. Session 9, *t*(21) = 2.10, *p* = 0.048; Site 3 Session 9 vs. Session 3, *t*(31) = 2.55, *p* = 0.016]. Site 1 did not show a single Session that clearly met criterion, with pairwise comparisons of Site 1 Sessions with the greatest UCH-L1 concentrations showing no difference (*p* values >0.05). Session 8 was used to represent post-blast exposure UCH-L1 concentrations for Site 1. Arrows indicate Sessions selected for comparison to baseline (Session 1).

**Figure 3:**
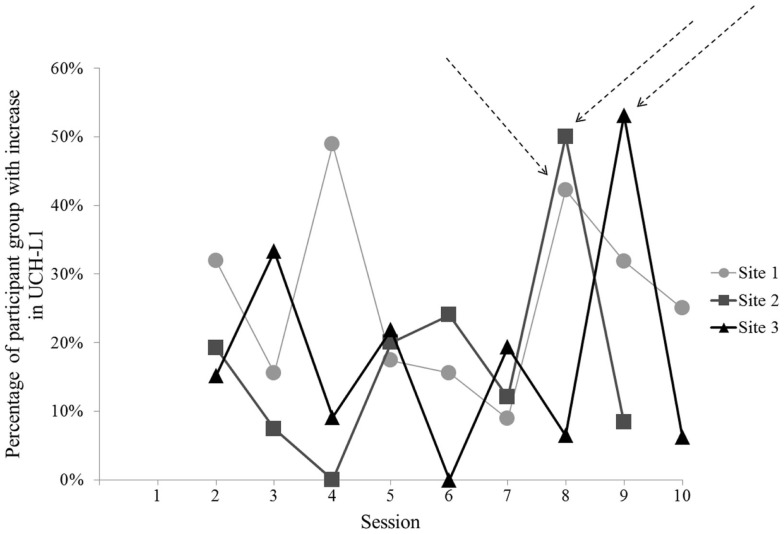
**The percentage of participants showing increase in normalized UCH-L1 from the previous day for each Session at each of the three sites**. Session 8 for Sites 1 and 2 and Session 9 for Site 3 show that 40% or more of the subjects showed an increase in UCH-L1. Arrows indicate Sessions selected for comparison to baseline (Session 1). No data are shown for Session 1 in this figure because there was no prior timepoint available to assess daily increase in UCH-L1.

For two of the three sites, the Session that met criterion for comparison to baseline (i.e., greatest UCH-L1 and group exposure to blast) clearly differed from the other Sessions (Figure 2). Site 1 did not have a single clear comparison day. To include Site 1 data in analyses, we selected Session 8 for comparison with baseline, due to the relatively high level of UCH-L1 measured, the increase in level relative to the preceding day [Site 1 Session 8 vs. Session 7, *t*(44) = 3.13, *p* = 0.003], and similarity of occurring in the second week of study (comparable to the Sites 2 and 3). For each of these Sites, the Session selected for comparison to baseline showed that >40% of subjects showed increase in UCH-L1 relative to the previous day (Figure 3), suggesting the best potential for examining a group-level phenomenon rather than an individual phenomenon.

**Figure 4:**
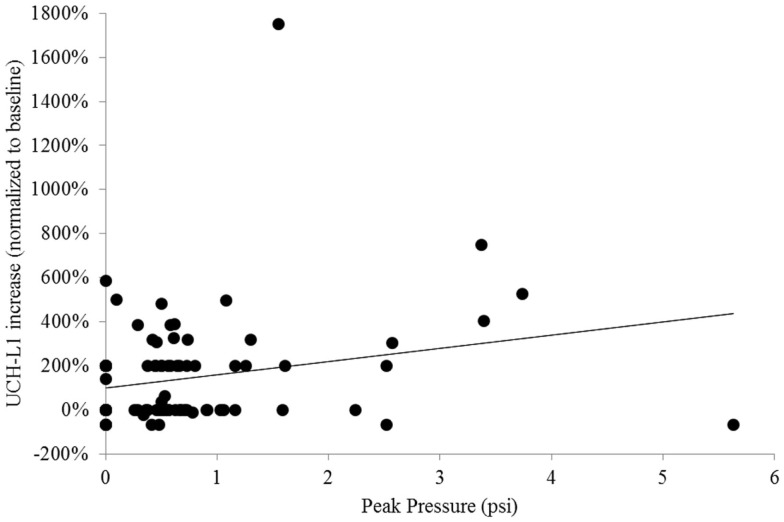
**Scatterplot of individuals’ normalized UCH-L1 from the Session that was compared to baseline in the primary analysis (Session 8 for Sites 1 and 2, Session 9 for Site 3) expressed as a function of the greatest peak overpressure recorded for that individual on that day**. The line represents simple linear regression.

In the primary analysis, we examined the association between UCH-L1 change and the greatest recorded peak pressure in blast exposures from Session 8 for Sites 1 and 2 and Session 9 for Site 3. To quantify the association between UCH-L1 concentration and magnitude of blast, we conducted a simple linear regression analysis, which yielded *R*^2^ = 0.05 and standard coefficient of 0.23 (*t* = 2.36, *p* = 0.020) (Figure 4). Six subjects were excluded from this analysis due to missing data. There was a reliable association between blast magnitude and UCH-L1 increase, but that association was not strong.

**Figure 5:**
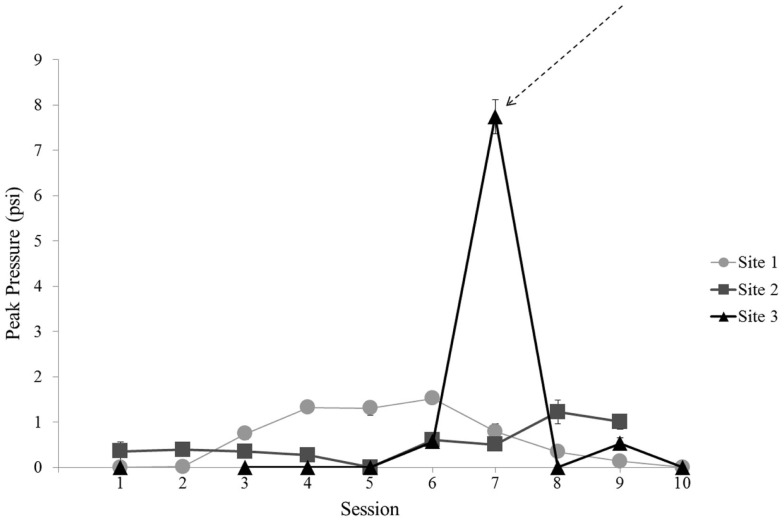
**Mean blast magnitude [peak pressure (psi)] recorded for each Session for each site**. Error bars are standard error. Data are not included for Session 2 at Site 3 due to the use of alternate recording equipment in that Session. The arrow indicates the unique Session selected for focused analysis of greatest blast magnitude on UCH-L1 concentration.

In a secondary analysis, baseline UCH-L1 was compared to UCH-L1 levels from the Session with exposure to the largest blast. The largest blast was recorded at Site 3 on the day of Session 7 (Figure 5). The other two sites did not have a comparable day of exposure so this analysis was conducted for Site 3 only. Of the 33 subjects enrolled at Site 3, 1 subject was not exposed to blast on Session 7 and was not included in this analysis. The remaining 32 subjects were exposed to peak pressures ranging from 5.0 to 11.7 psi. This exposure magnitude differed from the exposure in the preceding Session [*t*(31) = 23.98, *p* < 0.001]. Simple linear regression of UCH-L1 concentration and magnitude of blast for Session 7 showed no relation (*p* = 0.559).

**Figure 6:**
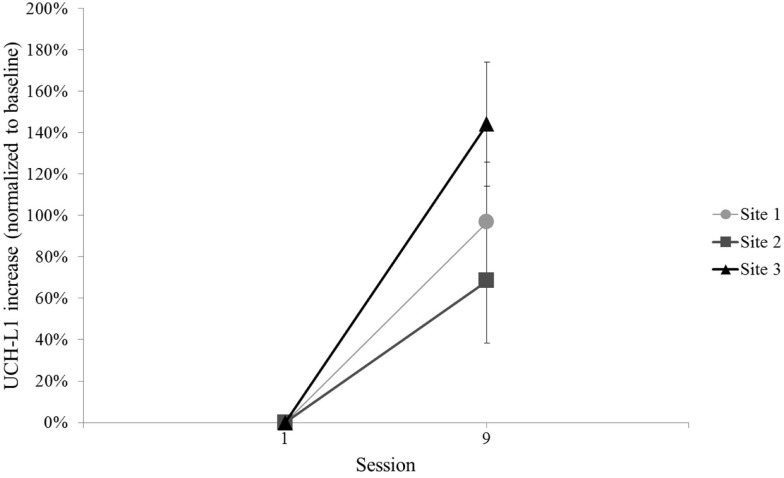
**Mean normalized UCH-L1 for baseline and the Session with the final blast exposure in the 2-week training protocol for each site (i.e., Session 9)**. Error bars are standard error.

The other secondary analysis of UCH-L1 relative to blast exposure considered all available blast exposures across the three sites by comparing the final Session with blast exposure to baseline, regardless of UCH-L1 concentration or blast magnitude recorded for that final Session. The final Session with blast exposure was Session 9 for each of the three sites (see Figure 1). This evaluation of UCH-L1 at Session 9 showed increased UCH-L1 [*t*(99) = 5.97, *p* < 0.001] (Figure 6). Two of the three sites included a final Session with no blast exposure (i.e., Session 10) and, thus, UCH-L1 measurements >24 h from blast exposure, which afforded an opportunity to examine UCH-L1 levels return to baseline when there was no acute exposure to blast. Comparison of Session 9 to Session 10 in those two Sites showed a decline in UCH-L1 concentration, *t*(75) = 2.53, *p* = 0.013.

**Figure 7:**
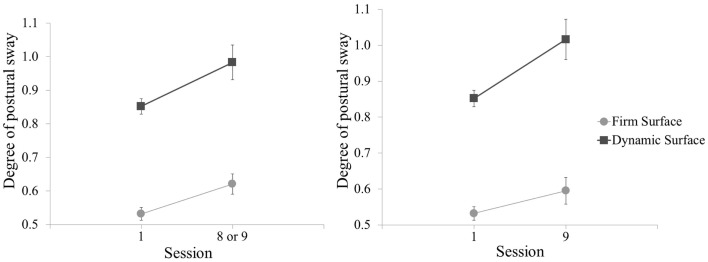
**Mean postural sway (standard deviation from center of gravity) for baseline and post-blast comparison Sessions, shown for test conditions with eyes open on each of two surfaces**. Greater degree of postural sway corresponds to worse performance. Left panel is baseline and the post-blast Session with the greatest UCH-L1 increase (Session 8 for Sites 1 and 2, Session 9 for Site 3). Right panel is baseline and the final post-blast Session (Session 9). All three sites are combined. Error bars are standard error.

For assessment of vestibular function corresponding to the Sessions compared in the primary analysis, the Session with the greatest level of measured UCH-L1 also showed increase in postural sway for the test conditions with eyes open, on both firm surface, *t*(97) = −2.76, *p* = 0.007 and dynamic surface, *t*(97) = −2.45, *p* = 0.016 (Figure 7, left panel). Postural sway with eyes open was also observed to increase in comparison of baseline to Session 9 (final blast exposure) but for dynamic surface condition only, *t*(96) = −3.34, *p* = 0.001 (Figure 7, right panel). The mean value increase for postural sway on firm surface did not meet statistical criterion. However, these increases in postural sway from baseline for the Sessions compared in the primary analysis and for Session 9 did not correlate with UCH-L1 increase in the same session (*p* values >0.05). There was also no association for postural sway in the eyes closed for firm surface or foam surface conditions.

In the supporting analyses of UCH-L1 and its association with magnitude of blast exposure, the symptomology, cognitive testing, and demographic data showed only one association. Among the 14 comparisons examined in the ANAM data (seven ANAM subtests, two timepoints), only the comparison between Session 9 (final blast exposure) UCH-L1 increase and ANAM Simple Reaction Time subtest performance decrease showed correspondence, *R*^2^ = 0.06 and standard coefficient of −0.25 (*t* = −2.43, *p* = 0.017). In the ANAM TBI Battery, the Simple Reaction Time subtest is administered twice and the second administration of the Simple Reaction Time subtest did not show this association, *R*^2^ = 0.03 and standard coefficient of −0.16 (*t* = −1.56, *p* = 0.123). This association was also not observed between UCH-L1 increase and ANAM Simple Reaction Time when Session 8 was considered for Sites 1 and 2 (the timepoints with the greatest UCH-L1 increase). The remaining ANAM subtests at the comparison timepoints showed no association with UCH-L1 or showed a positive relation (UCH-L1 increase corresponding to improvement in ANAM subtest performance). There were no associations between the blast-related UCH-L1 increases and DANA performance, self-reported symptomology, or demographic data. When UCH-L1 increase on the Session with maximum UCH-L1 or on Session 9 was compared to subject characteristics of age, history of head injury, blast exposure, or breaching, the correlations were low and did not meet criterion for statistical reliability (*p* values >0.05).

## Discussion

The purpose of this study was to examine UCH-L1 as a marker of neurological insult in individuals exposed to repeated low-level blasts. The results lend only limited support to the hypothesis that UCH-L1 measurements reflect blast-related environmental insult to the human CNS. Direct association to an acute effect from low-level blast was not clear, given unexplained outlier effects and the weakness of the observed association. Overall, these findings are consistent with a recent study of UCH-L1 in relation to sub-concussive sports-related impacts (12). Puvenna and colleagues observed increases in UCH-L1 in the context of participation in a contact sport (i.e., American football) but no association between UCH-L1 increases and measures of the degree of head impacts or other markers of CNS injury.

In the present study, when comparing baseline to the post-blast exposure Session with the greatest group-level UCH-L1 change, we found a correspondence between degree of UCH-L1 increase and greater magnitude of blast exposure, but there were clear deviations from that pattern. As exemplified in apparent outliers in Figure 4, there was an individual who exhibited more than 1000% increase from baseline in UCH-L1, yet the magnitude of his blast exposure was <2 psi and not apparently extraordinary. There was an individual who was exposed to blast of more than 5 psi (a high value in this study), yet he exhibited a decline in UCH-L1 concentration as compared to his pre-blast exposure baseline value. It seems likely that the simple association observed between blast and UCH-L1 in the aggregate is not capturing important factors. A factor of principal consideration is elapsed time between blast exposure and blood draw, which could not be controlled in this observational paradigm. The subject cited here with exposure to blast pressure >5 psi was available for blood draw 6 h 40 min following most recent exposure to blast. It may be that the trauma-related temporal dynamics of UCH-L1 are rapid enough that elapsed time is an essential variable for useful application of UCH-L1 as a marker of low-level blast exposure.

It also seems likely that there is an important contribution of prior blast exposure that occurs following a short-lived timeframe of UCH-L1 increase and decrease within 24 h of injury. When comparing baseline levels of UCH-L1 with levels after the final day with blast exposure in this 2-week paradigm regardless of blast magnitude that day UCH-L1 increases were observed. However, when comparing baseline levels of UCH-L1 to the Session with the largest single blast exposure, there was not an association between magnitude of blast and degree of UCH-L1 increase, at least not acutely. The total number of days of blast exposure may be more related to UCH-L1 increase than same-day magnitude of blast.

There was also an unclear correspondence between blast-associated increase in UCH-L1 and the behavioral data. Two of the four postural sway assessments showed results following blast exposure that were consistent with performance deficit. The other two assessments did not show such deficit, but it is relevant that those two conditions were the eyes closed conditions. Standing steady with eyes closed, regardless of supporting surface, is a novel activity for humans. As such, when challenged to do so everyday across a period of 10 days, people are likely to improve in their eyes closed skill. It is reasonable that a minor deficit in an eyes closed assessment of postural sway would be masked by a practice effect. In an eyes open condition, however, human subjects can be assumed to be at asymptote at baseline and post-blast assessments would be more sensitive to deficits in performance of the vestibular system. This interpretation of the postural sway results in this study is reasonable, but, even so, the degree of increase in postural sway following blast exposure did not correspond to degree of UCH-L1 increase, further diminishing inferences that can be drawn from UCH-L1 changes. The remaining behavioral data provided only one association with UCH-L1 to consider. The increase in blast-related UCH-L1 was associated with decreased performance on the ANAM Simple Reaction Time subtest in the final Session with blast exposure. In first consideration, this seemed relevant in that, of the ANAM subtests, the Simple Reaction Time subtest is regarded as of some clinical utility in assessment of concussion (25). However, there were notable inconsistencies in this association between blast-related UCH-L1 and cognitive reaction time, specifically in the parallel reaction time test within the same battery and the same Session (also shown to have clinical utility in assessment of concussion) (26), a similar assessment in the same Session (i.e., DANA Simple Reaction Time subtest), and the Simple Reaction Time subtest on the preceding day when larger blast-related UCH-L1 increase was observed. Further, in consideration of the 14 comparisons examined in the ANAM cognitive testing data, a correction to statistical criterion for multiple comparisons means that the single observed association may not be compelling. A finding of no clear relation between the behavioral data and UCH-L1 increase was somewhat expected, given that observation of change in behavioral performance in other studies has been rare. Field testing incurs more variability than clinical settings and can mask small cognitive changes. Therefore, we did not expect to find behavioral changes relevant to clinical diagnoses or operator performance of duties.

For these analyses of a neurotrauma biomarker among normal healthy undiagnosed individuals, it is reasonable that some subjects would not show any change in UCH-L1 concentration following exposure to low-level blast at any timepoint, which was the case in this study. The low levels of blast used in training environments do not yield injury, so, when change in UCH-L1 is observed, the key question is if that change is associated with blast or if that change more closely corresponds to some other variable. In these data, potential predictors of neurotrauma vulnerability and UCH-L1 change (age, history of head injury or blast exposure) did not meet statistical criterion to account for changes in UCH-L1 concentration; whereas magnitude of blast did. It is difficult to determine contributions of individual differences to expression of UCH-L1 as there has been no study of what constitutes normal levels in healthy young adults. Most human studies of UCH-L1 have been with brain injured patients (27). Additionally, based on the notion that sub-concussive behavioral symptoms after repeated exposures may accrue to produce injury-like symptoms is supported in populations that experience blast (4), one would expect a history of breaching to be differentiated by the biomarker. However, we observed no statistical difference in UCH-L1 levels between those with a history and those without. In the same way, UCH-L1 levels in individuals with a history of head injury were not different than levels in those with no history of head injury. These results suggest that while UCH-L1 may be a marker for acute clinical injury (14), it may not differentiate based on past sub-concussive or even concussive neurological insults.

This study was the first multi-site large sample human subjects study of an acute serum-based neurotrauma biomarker in conjunction with individualized measurement of concurrent blast magnitudes. There were, however, limitations resulting from the observational nature of the design and potentially from the selection of methods to measure and analyze blast magnitude. The approach to analysis maximized the likelihood that the predominant environmental exposure was blast overpressure, but unknown environmental factors could not be controlled and cannot be ruled out as influences on UCH-L1. For example, given that UCH-L1 is expressed by other cell types, such as neurons in the neuromuscular junction (28) and inflammatory cells (29), it is possible that serum increases observed are related to events other than neurological insult due to blast. Effects on the body globally may be related to rise in peripheral levels (12, 30).

Other biomarkers may be better candidates or important additions to assess acute effects of low-level blast exposure on the CNS, including biomarkers in cases of mild traumatic brain injury reported in this issue (Buonora et al., under review). These potential markers include: neuron-specific enolase (NSE) (31–33) and brain-derived neurotrophic factor (BDNF) (34, 35) as markers of neuronal damage and recovery; peroxiredoxin 6 (PRDX6) as an assessment of oxidative stress (36, 37); and S100 calcium binding protein beta (S100b) as a marker of glial damage and gliosis (33, 38). Future investigations of concurrent blood marker and blast exposure measurement should consider including these or other additional markers, as well as establishing normal levels in healthy young adults. Technological advances and new techniques can be expected to improve the assay of UCH-L1, especially at concentrations currently below assay thresholds, which may improve the ability to reveal associations with other parameters (such as blast exposure), However, the brain is a complex organ and it is reasonable that a multi-channel assessment will be more effective than a single channel assessment.

There are multiple methods to quantify blast exposure. Averaging peak pressure readings from sensors placed on the left and right sides of the helmet reduces the fidelity of the data. However, without accurate information on the orientation of the operator in relation to the blast, it is impossible to know how individual pressure sensor readings represent reflective and incident pressures. Such pressure differentiation becomes even more complex in settings with effective reflective surfaces and/or use of multiple charges in close proximity. Incident pressure readings are inherently lower than reflected, so by averaging the readings from the two sensors we are underestimating the overall pressure load to the head. In the high-paced, restricted training environments examined in this study, accurate logging of head orientation for all participants during all training events was not feasible. Therefore, the conservative estimates of blast exposure used in this analysis seemed most appropriate. Furthermore, other measured elements of the blast event, such as impulse energy, may have been superior predictors than peak pressure. However, different measures of blast pressure are positively correlated with each other and should yield similar variance across measures.

The objective in this study was to evaluate UCH-L1 as a serum-based marker of low-level blast exposure. Identifying such a serum-based biomarker will be particularly advantageous in military settings (5). The present study does not directly support serum-based UCH-L1 as an effective standalone marker for use in studies of subclinical effects from low-level blast. UCH-L1 may serve as a predictor of negative health effects from a larger magnitude blast than observed in the current study or as a contributory indicator among a panel of blood-based neurotrauma biomarkers. Alternately, it may be that UCH-L1 could serve as a standalone marker when assessed from cerebral spinal fluid (CSF) rather than serum (39), but CSF was outside the scope of the current study and not suitable as a routine assessment in a field setting. Our results do not diminish the promise of serum-based neurotrauma biomarkers in this domain, but further study of potential biomarkers is required.

## Conflict of Interest Statement

The authors declare that the research was conducted in the absence of any commercial or financial relationships that could be construed as a potential conflict of interest.

## Author Note

The views expressed in this article are those of the authors and do not necessarily reflect the official policy or position of the Department of the Army, Department of the Navy, Department of Defense, or the U.S. Government. MAJ Walter Carr, CPT(P) Angela M. Yarnell, COL Ricardo Ong, LT Uade da Silva, and CPT Matthew L. LoPresti are military service members and Dr. Gary H. Kamimori and Dr. Richard M. McCarron are federal civil service employees. This work was prepared as part of their official duties. Title 17 U.S.C. §105 provides that “Copyright protection under this title is not available for any work of the United States Government.” Title 17 U.S.C. §101 defines a U.S. Government work as a work prepared by a military service member or employee of the U.S. Government as part of that person’s official duties.
